# Light manipulation as a route to enhancement of antioxidant properties in red amaranth and red lettuce

**DOI:** 10.3389/fnut.2024.1386988

**Published:** 2024-06-05

**Authors:** Annika Bucky, Martina Pičmanová, Victoria Porley, Simon Pont, Ceri Austin, Tanveer Khan, Gordon McDougall, Alexandra Johnstone, Derek Stewart

**Affiliations:** ^1^The Rowett Institute, University of Aberdeen, Aberdeen, United Kingdom; ^2^Advanced Plant Growth Centre, The James Hutton Institute, Invergowrie, Dundee, United Kingdom; ^3^Intelligent Growth Solutions, Invergowrie, Dundee, United Kingdom

**Keywords:** vertical farming, light ratio, antioxidants, controlled environment agriculture, nutritive value enhancement

## Abstract

With the growing global population and climate change, achieving food security is a pressing challenge. Vertical farming has the potential to support local food production and security. As a Total Controlled Environment Agriculture (TCEA) system, vertical farming employs LED lighting which offers opportunities to modulate light spectrum and intensity, and thus can be used to influence plant growth and phytochemical composition, including antioxidants beneficial for human health. In this study, we investigated the effect of four red-to-blue light ratios of LEDs (R:B 1, 2.5, 5 and 9) on the growth and antioxidant components in red amaranth microgreens and red lettuce. Plant growth, total phenols, betalains, anthocyanins, vitamin C and antioxidant capacity (ferric reducing antioxidant power assay) were evaluated. A higher proportion of red light resulted in biometric responses, i.e., stem elongation in red amaranth and longer leaves in red lettuce, while the increase in the blue light fraction led to the upregulation of antioxidative components, especially total phenols, betalains (in red amaranth) and anthocyanins (in red lettuce). The antioxidant capacity of both crops was strongly positively correlated with the levels of these phytochemicals. Optimizing the red-to-blue ratio in LED lighting could be effective in promoting antioxidant-rich crops with potential health benefits for consumers.

## 1 Introduction

Global population projections estimate 9.7 billion people by 2050, possibly 10.4 billion in 2100 (1), and with that a major challenge in achieving food security. Food production, consumption and waste are the key factors in shaping the health of people and the planet (2). Unhealthy diets pose a threat for people and the planet on a global scale. Even though the global food system contributes 21–37% (3) of global greenhouse gas emissions, there is still a high prevalence of malnutrition and food insecurity. Poor diets are a significant burden on the global health system and society (4). The combination of population growth, limited, arguably diminishing, farmland available for food production and the impacts of climate change, have raised interest in Controlled Environment Agriculture (CEA), and particularly vertical farming, as a robust route to the production of some foodstuffs (5). These systems offer the potential for continuous production (24/7/365), albeit with a requirement for energy inputs for temperature, humidity, and lighting control facilitating the delivery of fresh and nutritious food available at national and local levels. The extension of CEA to vertical farming takes the concept further as it reduces the growing system footprint by growing in three dimensions and as a result is opening up the opportunity of urban food production using derelict and/or poorly used land (6, 7).

Light is arguably the most essential factor for plant growth and development, and the controlled lighting systems in vertical farming are being utilized to influence plant growth rates, yield and composition including important nutrients and health-beneficial phytochemicals (8–10). Light-emitting diodes (LED) lights have been used as an efficient light source for commercial plant cultivation in vertical farming. They provide the opportunity to modulate light quality (wavelengths), intensity and duration (photoperiod), which play a key role in plant physiological processes (11). Plants use wavelength-specific photoreceptors known as phytochromes (red/far red light), cryptochromes (blue light/UV-A), and phototropins (blue light) to perceive and interpret incoming light signals to regulate their physiology and development (12, 13). Currently, red light (approx. 600–700 nm) and blue light (approx. 400–500 nm) have been recognized as the most suitable treatments for plant growth and development of tailored food (11), although other wavelengths, e.g., UV, are well reported to enhance secondary metabolites such as (poly)phenolics (13, 14). Indeed, these secondary metabolites and some primary metabolites comprise a major component of the plant antioxidant system. These compounds are produced in plants as part of their defense mechanisms in response to various environmental stresses and/or to play a crucial role in neutralizing reactive oxygen species (ROS) and maintaining a steady oxidative state to allow normal plant biochemical function (15). Many of these components have also been reported to exert health benefits when eaten by humans (16, 17).

The antioxidant effect of plant foods is mainly attributed to compounds such as flavonoids, including anthocyanins, phenolic acids, tannins, betalains, carotenoids, glucosinolates and certain vitamins (18–20). It has been shown that the consumption of plant foods high in antioxidants (rather than isolated supplements) is associated with a lower risk of chronic oxidative stress and the related symptoms (21–23). The biosynthesis of antioxidants in plants can be influenced by light intensity and light spectrum; for instance, anthocyanin levels increased in red holy basil but decreased in mustard microgreens when blue light only or higher blue light fractions were used (24, 25).

Among the most common crops grown in vertical farms are salad crops and microgreens. Microgreens are tender immature greens grown from the seeds of vegetables and herbs, harvested upon the appearance of the first pair of true leaves when the cotyledons are fully expanded (26). Recent studies have revealed that microgreens are richer than mature greens in some vitamins, sugars, and antioxidants, including carotenoids (27). The access to this new form of nutrient dense produce provides a great opportunity to deliver micronutrients and phytochemicals in a safe way. In addition, red/darker varieties of vegetables have higher nutrient composition, anthocyanin content, vitamin C, and mineral content compared to green vegetables (19, 28, 29). Thus, two crops of red/darker variety were chosen for our study: red amaranth microgreens and red lettuce. For their high content of nutrients, including the antioxidants betalains and anthocyanins, respectively (19, 28, 30), these crops are gaining great popularity as a healthy and sustainable food option. In this study, red amaranth and red lettuce were grown in a vertical farm under different red-to-blue ratios (R:B) of the LED light spectrum, with the goal to examine the effect of the light treatments on growth and on the content of phytochemicals with antioxidant capacity.

## 2 Materials and methods

### 2.1 Plant materials and growing conditions

The experiment was conducted in a 10-week period in the state-of-the-art vertical farm at the Intelligent Growth Solutions Ltd. Crop Research Center in Invergowrie, Scotland. The seeds of Micro Amaranth Red Aztec (*Amaranthus tricolor* L.) and Red Batavia Lettuce (*Lactuca sativa* L.) were purchased from CN seeds (Ely, United Kingdom) and were sown using a Mosa Drum Seeder sowing machine (Mechanical Botanical, Chiddingfold, United Kingdom) into propagation trays filled with a substrate medium produced from a 75:25 (v/v) mix of coconut coir and vermiculite. The propagation trays were then transferred into a dark germination chamber for 2 days and then placed on the growth trays with lights (GTLs) in the vertical farm growth towers.

LED lights (Osram, Austria) were arranged horizontally 300 mm above the tray base in the GTLs and emitted blue light (B, λ = 400 nm-499 nm, peak at 451 nm), green light (G, λ = 500 nm-599 nm, peak at 521 nm), red light (R, λ = 600 nm-699 nm, peak at 660 nm) and far-red light (FR, λ = 700 nm-799 nm, peak at 730 nm). The crops were grown under four different light regimes: the red-to-blue light ratio (R:B) was adjusted to 1, 2.5, 5 and 9 (Table 1). The photosynthetic photon flux density (PPFD) was kept as close to the standard light recipe (2.55) as possible at ~255 μmol m^−2^ s^−1^ (Table 1). The photoperiod was 18 h d^−1^, temperature 25°C, relative humidity 70–75%, pH and EC of the nutrient solution were 5.7–6.3 and 1.7–1.8 dS m^−1^, respectively; the nutrient solution and watering schedule were kept identical for all the light treatments.

**Table 1:** Four light recipes used for the treatment of red amaranth and red lettuce in the vertical farm.

Treatment	R:B ratio	Blue (μmol)	Green (μmol)	Red (μmol)	Far red (μmol)	PPFD (μmol m^−2^ s^−1^)
RB1	1.01	111.0	12.5	112.5	17.0	253.0
		44%	5%	44%	7%	
RB2.5	2.55	63.6	12.5	162.2	17.0	255.3
		25%	5%	63%	7%	
RB5	5.05	37.2	12.5	187.8	17.0	254.5
		15%	5%	73%	7%	
RB9	9.09	22.3	12.5	202.6	17.0	254.4
		9%	5%	79%	7%	

R:B ratio refers to the ratios of light intensity of red and blue light fractions.

Plants were harvested nine and 22 days after sowing for amaranth and lettuce, respectively. The leaf length in lettuce was recorded as well as the stem height in red amaranth. After the fresh weight was recorded, the plants were snap frozen in liquid nitrogen and freeze-dried using Christ Gamma 1–16 LSC freeze dryer (Osterode am Harz, Germany) for a minimum of 3 days. The dry matter was weighed to determine the percentage of dry weight and the plant material was homogenized using Retch ZM 200 ultracentrifugal mill with sieve size 0.5 mm (Haan, Germany). Samples were kept in 50 mL-falcon tubes at −20°C until required for analysis. Every biological replicate represented a pool of two propagation trays.

### 2.2 Extract preparation

For colorimetric analyses, 10 mL of 80% methanol was added to 200 mg of homogenized plant material in a 15 mL falcon tube. The suspensions were vortexed for 30 s, sonicated in an ultrasonic bath with ice for 15 min then agitated on an orbital shaker for 30 min. The samples were then centrifuged for 10 min at 3,900 rpm, and the supernatants stored at −80°C.

For vitamin C analysis, 50 mg of homogenized plant material in a 2 mL microtube was resuspended in 1.5 mL of 5% (w/v) metaphosphoric acid containing 5 mM tris(2-carboxyethyl)phosphine (TCEP). The suspension was vortexed for 10 s and agitated on IKA Vibrax VXR basic shaker at 1,750 rpm for 60 min at 5°C. The suspension was then centrifuged at 5°C for 10 min at 13,000 rpm and the supernatant filtered in Thomson Standard Filter Vials prior analysis.

### 2.3 Chemical analysis

#### 2.3.1 Total polyphenols

Total phenolic content (TPC) was measured using the method described by Slinkard and Singleton (31). Amaranth and lettuce methanolic extracts were diluted with distilled water to 20 and 10% solutions, respectively. To 250 μL of diluted samples, 250 μL of Folin–Ciocalteu reagent was added and the mixture incubated for 3 min at room temperature. Further, 500 μL of saturated sodium carbonate were added and the samples incubated for 1 h at room temperature. The absorbance was measured using Ultrospec 2100 spectrophotometer (Amersham Biosciences, Buckinghamshire, United Kingdom) at 750 nm. Quantification was made using the gallic acid standard curve (0.02–0.8 mg mL^−1^, *r*^2^ = 0.9918). Results were expressed in g of gallic acid equivalent (GAE) per 100 g of sample on dry weight (g GAE 100 g^−1^ DW) and fresh weight basis (g GAE 100 g^−1^ FW).

#### 2.3.2 Betalains

Betalains were measured in red amaranth only as they are absent from red lettuce (32). The quantification of betalains (as sum of betacyanins and betaxanthins) was carried out according to Sokolova et al., with minor modifications (33). Methanolic extracts were diluted with water to 10% solutions and the content of betacyanins and betaxanthins was determined at 536 and 485 nm, respectively, using the following equation:


$$\text{Betalains}\;\left(\text{mg}\;L^{-1}\right)=\frac{A\times MW\times DF\times 10^{3}}{\varepsilon \times l}$$

where A is optical density in nm (for betacyanins: A = A_536nm_ − A_650nm_, and for betaxanthins: A = A_485nm_ − A_650nm_); DF = dilution factor; MW = molecular weight (550 g mol^−1^ for betacyanins, and 339 g mol^−1^ for betaxanthins); ɛ = molar extinction coefficient in L mol^−1^ cm^−1^ (60,000 for betacyanins, and 48,000 for betaxanthins); l = path length in cm; 10^3^ = factor for conversion from g to mg; the measurement at the wavelength of 650 nm was used to correct for impurities. Total betalains were calculated by adding betacyanins and betaxanthins and the results were expressed as mg betalains per 100 g of sample in dry weight (mg 100 g^−1^ DW) and fresh weight (mg 100 g^−1^ FW).

#### 2.3.3 Anthocyanins

Anthocyanins were measured in red lettuce only as they are absent from red amaranth (34). The quantification of anthocyanins was carried out using a pH differential absorbance method according to Lee et al. (35) with minor modifications. Solutions of 0.2 M potassium chloride (pH 1.0) or 0.1 M sodium acetate (pH 4.5) were added to the methanolic extracts in a 9:1 ratio, mixed and incubated for 10 min at room temperature. The absorbance was measured using a spectrophotometer at 510 nm and 700 nm in buffers at pH 1.0 and pH 4.5, respectively. The concentration of anthocyanins was estimated using the following equation:


$$\text{Anthocyanins}\;\left(\text{mg}\;C3G\;\text{equivalents}\;L^{-1}\right)=\frac{A\times MW\times DF\times 10^{3}}{\varepsilon \times l}$$

where A (optical density in nm) = (A_510nm_ – A_700nm_) pH 1.0 – (A_510nm_ – A_700nm_) pH 4.5; MW (molecular weight) = 449.2 g mol^−1^ for cyanidin-3-glucoside (C3G); DF = dilution factor; ε = molar extinction coefficient, in L mol^−1^ cm^−1^ (26,900 for C3G); l = path length in cm; 10^3^ = factor for conversion from g to mg.

The quantity of anthocyanins was expressed as mg of C3G equivalents per 100 g of sample on dry weight (mg C3GE 100 g^−1^ DW) as well as fresh weight basis (mg C3GE 100 g^−1^ FW).

#### 2.3.4 Vitamin C

Vitamin C (ascorbic acid) content was determined as described previously by Freitag et al. (36). Briefly, the samples were analyzed using HPLC (ASI-100 autosampler, and Ultimate 3000 pump) coupled to a DAD detector (UVD340U, Dionex, ThermoFisher Scientific, United Kingdom). Sample (20 μL) was injected onto an ICSep COREGAL-64H column (7.8 × 300 mm × 10 μm, ChromTech, United States) and an isocratic run of 30 min with a mobile phase containing 4 mM sulfuric acid in ultrapure water was applied. Ascorbic acid was quantified at 245 nm against external calibration curve of ascorbic acid (75–250 μg mL^−1^, *r*^2^ = 0.999) and the results expressed as both mg 100 g^−1^ DW and mg 100 g^−1^ FW.

#### 2.3.5 Antioxidant capacity

Ferric reducing antioxidant power assay (FRAP) was used to measure antioxidant capacity (37). In short, 900 μL of FRAP reagent consisting of 300 mM sodium acetate trihydrate (pH 3.6), 10 mM ferric 2,4,6-tripyridyl-s-triazine (TPTZ) in 40 mM hydrochloric acid and 20 mM ferric (Fe^3+^) chloride hexahydrate in a 10:1:1 ratio, was added to 100 μL of diluted methanolic extracts (10% solution of amaranth extract; 5% solution of lettuce extract). The absorbance was measured at 593 nm using a spectrophotometer after exactly 4 min following the addition the FRAP reagent. The reaction was estimated based on the ferrous (Fe^2+^) sulfate standard curve (100–1,000 μM, *r*^2^ = 0.998). Results were expressed as mmol Fe^2+^ 100 g^−1^ sample on dry weight (mmol Fe^2+^ 100 g^−1^ DW) and fresh weight basis (mmol Fe^2+^ 100 g^−1^ FW).

### 2.4 Statistical analysis

Statistical analysis was conducted with IBM SPSS Version 29.0.1.0 (171; SPSS Inc., United States). The results shown are the means of five biological replicates ± standard deviation (SD). One-way analysis of variance (ANOVA) at significance level of *p* < 0.05 with Tukey’s HSD *post-hoc* test was used to determine statistical differences between light treatments. Pearson correlation analysis was performed using the MetaboAnalyst 5.0 web-based platform (38).

## 3 Results

### 3.1 Growth and weight

**Figure 1:**
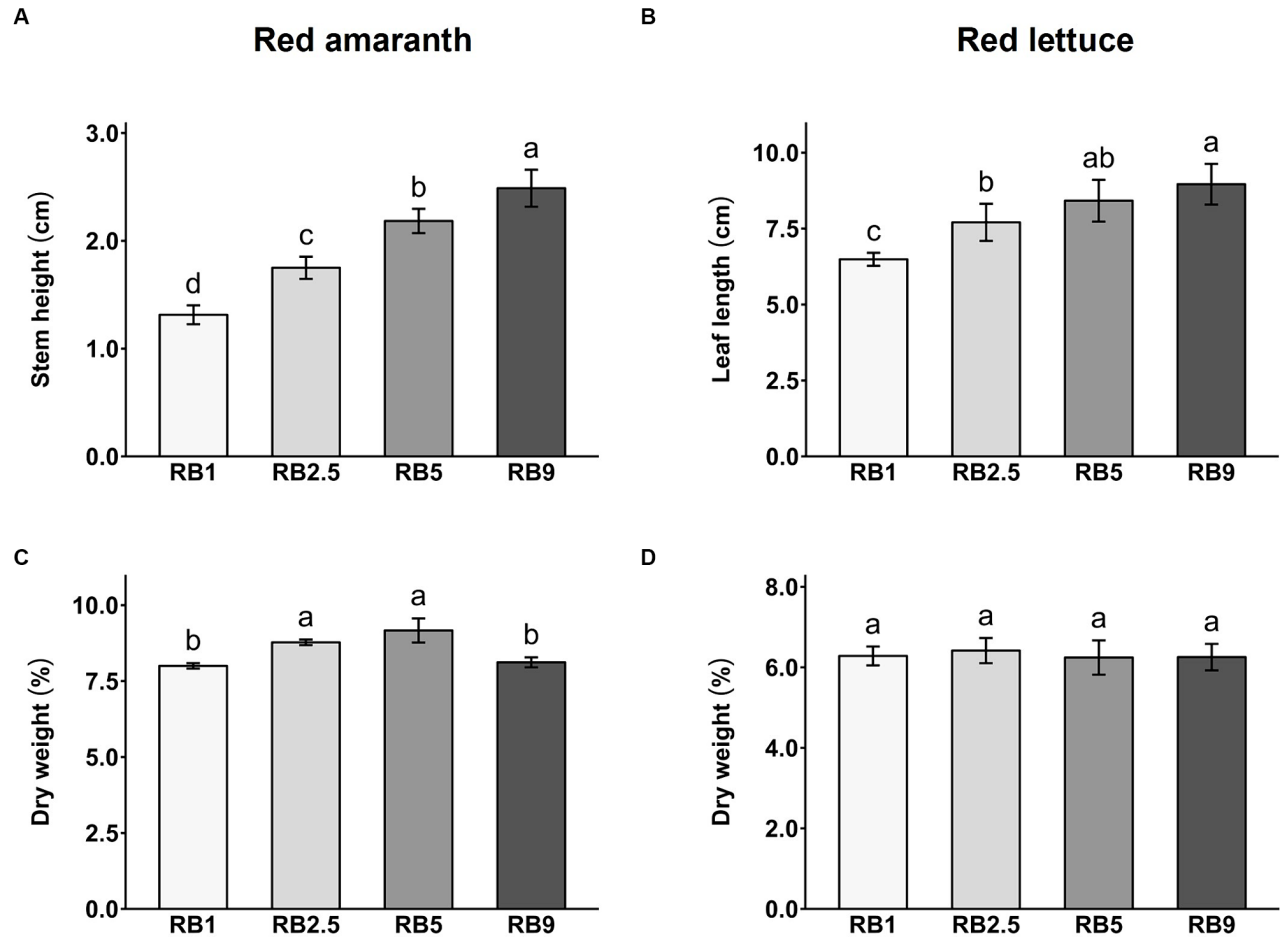
Stem height of red amaranth **(A)** and leaf length of red lettuce **(B)**, dry weight percentage of red amaranth **(C)** and red lettuce **(D)** affected by four light ratios. Values represent mean ± SD (*n* = 5). Different letters indicate significant difference among treatments for each crop (ANOVA, Tukey’s test, *p* < 0.05).

The effect of different light treatments on the dry biomass content and growth of red amaranth and red lettuce was evaluated. The light treatments significantly affected stem height and leaf length in red amaranth and red lettuce, respectively (Figures 1A,B; Supplementary Table S1). As the proportion of red light increased, the stem height in amaranth significantly increased, with the RB9-treated plants being the tallest (Figure 1A). A similar trend was observed for the leaf length in lettuce. Higher R:B ratio resulted in longer leaves, with the longest under RB9 treatment (Figure 1B; Supplementary Table S1). RB1-treated plants of both species were significantly shorter in height or length when compared to all other treatments. In red amaranth, the percentage of dry weight (DW%) was significantly higher in RB2.5- and RB5-treated plants (8.79 and 9.17%, respectively) compared to RB1 and RB9 treatments (7.98 and 8.12%, respectively; Figure 1C; Supplementary Table S1). In red lettuce, there were no significant differences between treatments in DW%, with the average value of 6.29% DW (Figure 1D; Supplementary Table S1).

### 3.2 Antioxidant components and antioxidant capacity

**Figure 2:**
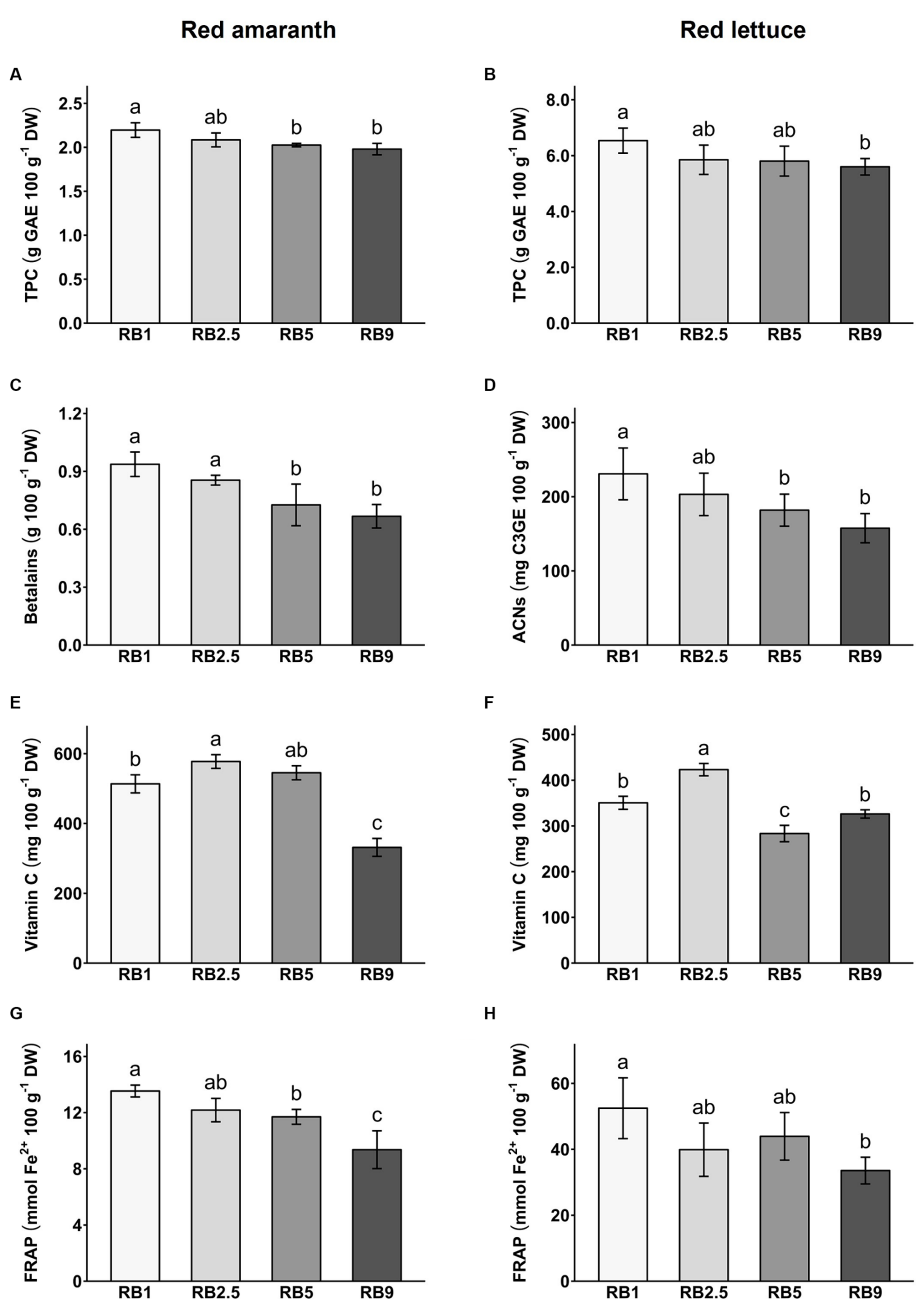
Total phenol content (TPC; **A** and **B**), betalains/anthocyanins (ACNs; **C** and **D**), vitamin C (**E** and **F**) and FRAP antioxidant capacity (**G** and **H**) in red amaranth and red lettuce under four different light treatments. Values represent mean ± SD (*n* = 5). Different letters indicate significant difference (ANOVA, Tukey’s test, *p* < 0.05).

To evaluate the antioxidant content in red amaranth and red lettuce, total phenol content (TPC), total betalains (in red amaranth), total anthocyanins (in red lettuce), vitamin C and antioxidant capacity (FRAP) were quantified per dry weight (DW; Figure 2; Supplementary Table S2) and fresh weight (FW; Supplementary Table S3).

### 3.3 Total phenol content

The different light recipes had a significant effect on total phenol content in red amaranth and red lettuce (Figures 2A,B). The TPC ranged from 1.98 to 2.2 g GAE 100 g^−1^ DW in red amaranth and from 5.6 to 6.54 g GAE 100 g^−1^ DW in red lettuce. In red amaranth, RB1-treated plants had significantly higher content of total phenols than RB5- and RB9-treated plants. In red lettuce, RB1-treated plants were significantly higher in total phenols than plants treated with RB9. In both plant species the trend was generally similar toward reduced TPC with increasing red/blue light ratio.

### 3.4 Betalains and anthocyanins

In red amaranth, the light recipes tested had a significant effect on betalain content (Figure 2C). It ranged from 667 to 937 mg 100 g^−1^ DW. RB5- and RB9-treated plants were lower in betalains than plants under other light treatments and the recipe with the highest blue light fraction (RB1) resulted in the highest betalain content. In red lettuce, total anthocyanins ranged from 158 to 231 mg C3GE 100 g^−1^ DW. Anthocyanins were significantly lower in RB5- and RB9-treated plants compared to the RB1 treatment (Figure 2D). The highest blue light fraction (RB1) resulted in the highest anthocyanin content. As for the TPC, the trend was for a reduced betalain and anthocyanin content with increasing red/blue light ratio.

### 3.5 Vitamin C

The vitamin C content ranged from 331 to 577 mg 100 g^−1^ DW in red amaranth and from 283 to 423 mg 100 g^−1^ DW in red lettuce (Figures 2E,F). The different R:B ratios had a significant effect on vitamin C content in both crops. In red amaranth, RB2.5 light recipe resulted in a significantly higher vitamin C content than RB1 and RB9 recipes. RB9-treated plants had significantly lower vitamin C content compared to the other treatments (Figure 2E). In red lettuce, RB2.5 led to significantly higher levels of vitamin C compared to all other treatments, while RB5-treated plants had significantly lower vitamin C content compared to the other treatments (Figure 2F). Therefore, no clear trend of vitamin C accumulation with red/blue light ratio was evident.

### 3.6 Antioxidant capacity (FRAP)

The light recipes had a significant effect on antioxidant capacity in both red amaranth and red lettuce (Figures 2G,H). The antioxidant capacity ranged from 9.4 to 13.5 mmol Fe^2+^ 100 g^−1^ DW in red amaranth and from 33.6 to 52.5 mmol Fe^2+^ 100 g^−1^ DW in red lettuce. In both crops, RB9-treated plants had significantly lower antioxidant capacity than plants grown under the other light treatments and the treatment with the highest blue light fraction resulted in the highest antioxidant capacity. This trend was also observed for TPC and betalain content with a decreasing value as the red/blue light ratio increased, which suggests that (poly)phenols and betalains make dominant contributions to the antioxidant capacity.

### 3.7 Correlations

**Figure 3:**
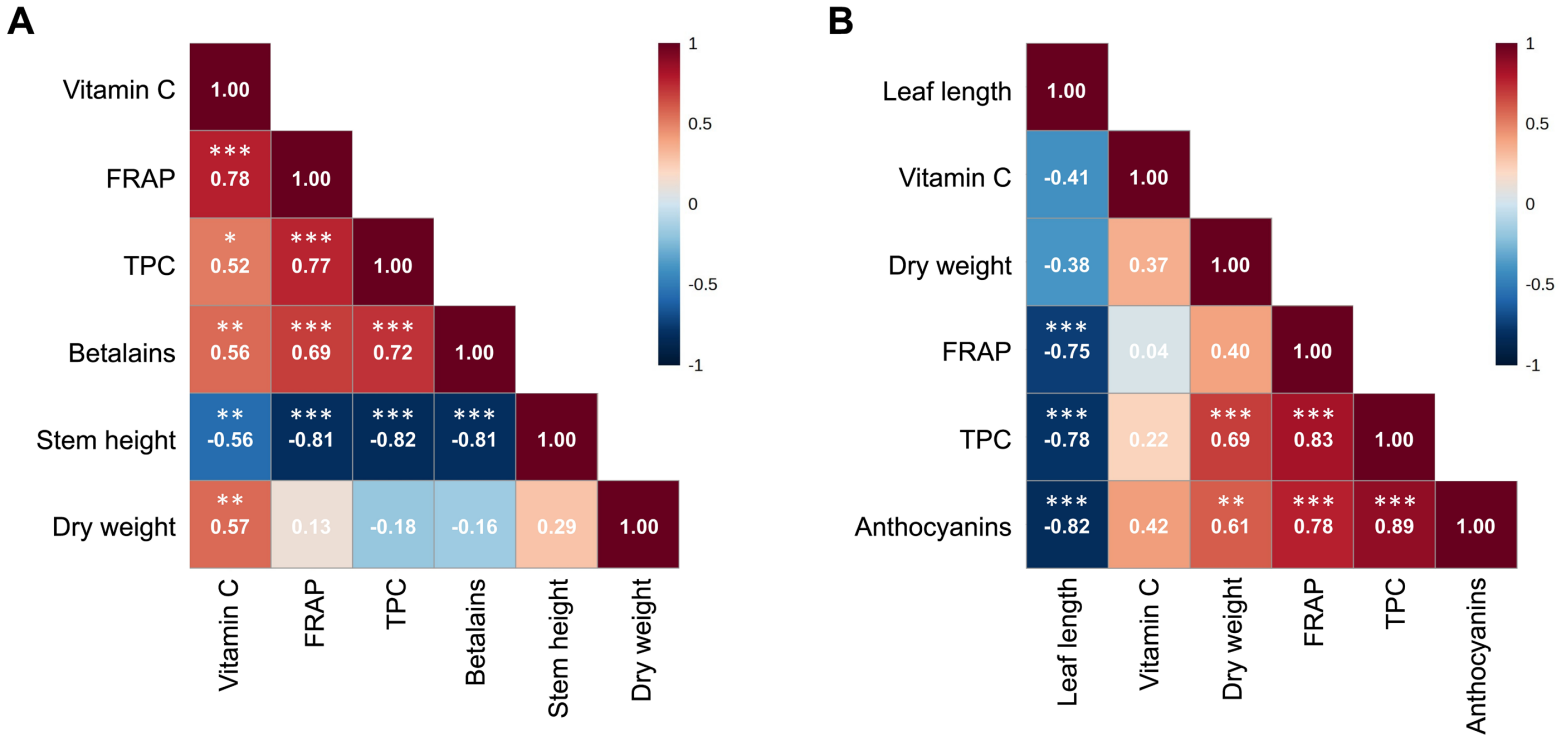
Correlation heatmaps showing Pearson correlation coefficients between stem height/leaf length, dry weight percentage, total phenol content (TPC), betalains/anthocyanins, vitamin C and FRAP in dry weight of red amaranth **(A)** and red lettuce **(B)**, as generated by MetaboAnalyst 5.0. Data were log_10_-transformed and auto-scaled; *p < 0.05, **p < 0.01, ***p < 0.001.

In red amaranth, correlation analysis (Figure 3A) revealed a strong positive correlation between FRAP and vitamin C (*r* = 0.78, *p* < 0.001), FRAP and betalains (*r* = 0.69, *p* < 0.001), FRAP and TPC (*r* = 0.77, *p* < 0.001) and between TPC and betalains (*r* = 0.72, *p* < 0.001). Moderate positive correlations were observed between vitamin C and betalains (*r* = 0.56, *p* < 0.01), vitamin C and TPC (*r* = 0.52, *p* < 0.05) and vitamin C and dry weight (*r* = 0.57, *p* < 0.01). Stem height was strongly negatively correlated with FRAP (*r* = −0.81, *p* < 0.001), betalains (*r* = −0.81, *p* < 0.001) and TPC (*r* = −0.82, *p* < 0.001). In red lettuce (Figure 3B), a strong positive correlation was found between TPC and anthocyanins (*r* = 0.89, *p* < 0.001). FRAP was strongly positively correlated with TPC (*r* = 0.83, *p* < 0.001) and anthocyanins (*r* = 0.78, *p* < 0.001). Dry weight was strongly or moderately correlated with TPC (*r* = 0.69, *p* < 0.001) and anthocyanins (*r* = 0.61, *p* < 0.01). Leaf length was strongly negatively correlated with FRAP (*r* = −0.75, *p* < 0.001), TPC (*r* = −0.78, *p* < 0.001) and anthocyanins (*r* = −0.82, *p* < 0.001). Vitamin C was not significantly correlated to any other parameter. The correlation heatmaps for the same parameters quantified in fresh weight are shown in Supplementary Figure S1.

## 4 Discussion

The present study indicates that the R:B ratio of LED lighting has a significant effect on the growth and antioxidant content in red leafy vegetables grown in a vertical farm. Physical characteristics such as stem height and leaf length are commercially important for growing produce to make it more appealing to consumers. In previous studies, both significant and non-significant responses in yield to different R:B ratios have been shown in various crops including red and green lettuce, sprouting broccoli and basil (39–43). In several lettuce cultivars red illumination increased growth and yield (39, 43). An increase in leaf area has also been achieved in lettuce cultivars by using increased red light proportion (44–46), which is consistent with our study in terms of higher red light fraction resulting in plants with longer leaves. Higher blue light percentages (lower R:B ratio) decreased plant height in *Brassica* microgreens and green holy basil (25, 47) whereas Rihan et al. reported an increased growth and yield in sweet basil with lower R:B ratios (41). Green and far-red light also play a role in the response of the plants to the light. The addition of green light had a positive and significant impact on leaf growth and yield in lettuce (39). In our study, the total quantity of green and far-red light fractions was kept constant in all treatments, but we recognize that their ratio to red and blue fractions varied in the four tested light recipes, which could have an additional effect on the measured parameters. Our results suggest that the increase in R:B ratio is favorable for red amaranth and red lettuce plant growth, regarding stem height and leaf length, respectively. However, this effect might not be always desirable as very long stalks or leaves might reduce marketability in a commercial setting, although the ability to vary this has been shown to be useful as a global/regional preference in the same crop criteria can vary considerably (48). Moreover, our data indicate that red amaranth with higher stems and red lettuce with longer leaves under higher R:B ratio have significantly lower levels of antioxidants and thus also reduced potential health beneficial effects for consumers (which, in the case of red lettuce, could possibly be caused by a dilution effect, as positive correlations were observed between dry weight and TPC/anthocyanins).

Phenolic compounds such as flavonoids and phenolic acids are plant metabolites that help protect plants against biotic (e.g., pathogens) and abiotic stressors (e.g., drought, high amount of light). There is also an extensive literature on the health benefits of phenolic compounds, including their cytotoxic, anti-inflammatory, antihypertensive, and anti-diabetic actions (49, 50). Blue light has been shown to enhance the accumulation of phenolic compounds in certain species (13, 25). In the present study, the increase of the proportion of blue light (lower R:B ratio) led to a higher content of total phenols (TPC) in both red amaranth and red lettuce. This is consistent with the findings by Kwack et al., showing that higher red-light intensity resulted in reduced total phenolic content in alfalfa sprouts and red radish (51). In Chinese kale sprouts, monochromatic blue light has been shown to significantly increase total phenol content compared to darkness, while monochromatic red light had no effect on TPC (52). A lower R:B ratio has been shown to increase total phenolics in lettuce and basil (25, 46). Thus, blue light can be used to increase total phenols in a variety of crops, but consideration is needed as the effects are species dependent and species respond differently in terms of phytochemical accumulation (53). Moreover, the addition of UV-A and UV-B light to the spectrum might result in enhanced accumulation of phenolic compounds with antioxidant properties as was demonstrated, e.g., in lettuce (54).

Secondary metabolites also play a role in taste, color and aroma of plants (13). Betalains (indole-derived chromoalkaloids typical for Caryophyllales) and anthocyanins (class of flavonoids) commonly give plants their characteristic red, purple, or yellow color. Anthocyanins and betalains are mutually exclusive, with the latter largely replacing the former in plants of the order Caryophyllales that also includes the genus *Amaranthus* (55, 56). In the present study, the highest concentration of betalains in red amaranth was detected under the highest blue fraction (RB1) and, as in the case of the phenolics, decreased with the increase in red light fraction. It was shown previously that red amaranth treated with blue light compared to dark conditions, had increased levels of betalains due to the upregulation of key genes involved in the synthesis of betalains by blue light (56). Similarly, the increase in the proportion of blue light led to significantly higher levels of anthocyanins in red lettuce, which is consistent with the previous light studies on red lettuce (46, 57). This is likely to be related to blue light activating the expression of genes that induce anthocyanin biosynthesis (57). In Chinese kale sprouts, monochromatic blue light irradiation has been shown to be the most effective in increasing anthocyanin content compared to other light treatments (52). The strong correlation of antioxidant capacity with betalains and anthocyanins indicates that they play an important role in the antioxidant capacity of red amaranth and red lettuce, respectively. Future work could examine if these changes in total betalain and anthocyanin contents are accompanied by changes in the profiles of the individual betalains/anthocyanins and other phytochemicals and elucidate the precise mechanisms underlying the complex signaling networks that regulate plant biochemical processes in response to different red and blue light ratios (including the regulation of the tyrosine-based betalain biosynthetic pathway and the phenylpropanoid pathway). Enhanced blue light was previously shown to increase flavonoids and phenolic acids in red leaf lettuce, including quercetin-glycosides, protocatechuic acid and chicoric acid (53). On contrary, Lee et al. found that supplemental red but not blue irradiation in red lettuce led to significantly enhanced accumulation of individual phenolic compounds, including chlorogenic acid, caffeic acid, chicoric acid, rutin, kaempferol, and luteolin, while the total phenol content was higher under the supplemental blue light (58). Other pigments than betalains/anthocyanins that could have been affected by the R:B treatments, but were not measured in this study, are chlorophylls and carotenoids. A one-to-one ratio of red and blue light seems to be the most effective treatment to achieve higher levels of chlorophyll contents in red amaranth and red lettuce (39, 45, 59–61), as blue light addition is key to chlorophyll biosynthesis in plant leaves (62, 63). Carotenoids have been shown to be increased under blue light in red amaranth and lettuce (60, 61, 64), which correlates with our study findings as they are accessory pigments related to antioxidant mechanisms (65).

Vitamin C (ascorbic acid) is an essential dietary vitamin required as a co-factor for many enzymes and can act as an important antioxidant in many body tissues (66). It plays a key role in bodily processes, e.g., iron absorption, collagen synthesis and immune system stimulation (67, 68). Light influences vitamin C accumulation in plants and is dependent on exposure time as well as intensity and quality of light (11, 67). Here, the RB2.5-treated plants had the highest levels of vitamin C in both crops. There was no clear trend observed in the vitamin C levels in relation to the R:B ratio. In previous studies on lettuce, the highest content of vitamin C was detected under 100% red light, indicating that pure red light can promote the synthesis or accumulation of vitamin C in lettuce most probably as a result of light stress (69, 70). In our study, the levels of vitamin C in both crops reacted to an increase in blue light as well as red light. The literature has shown both increases and decreases in vitamin C content in plants treated with higher levels of blue or red light (71–73). In kale sprouts the highest levels of vitamin C were found in plants under white LED treatment, followed by red LED treatment (52). In our study, vitamin C correlated strongly with antioxidant capacity in red amaranth. However, there was no significant relationship found for vitamin C in red lettuce, indicating that the high amounts of phenolic compounds present in red lettuce were the main contributors to the antioxidant capacity (45).

In both crops, the treatment with the highest proportion of blue light (RB1) resulted in the highest antioxidant capacity. Blue light has been previously shown to increase antioxidant capacity in lettuce (45, 74). The effects of light spectrum on total phenol content and betalains/anthocyanins are reflected in the total antioxidant capacity in both crops, with significant positive correlations in both red amaranth and red lettuce. Phenolic compounds are considered important antioxidants as they act as free radical scavengers (17, 75). Previously, a positive correlation between antioxidant capacity and TPC was demonstrated, for example, for basil cultivars and sage (25, 76). Similarly, betalains are known as very strong antioxidants, even superior to many flavonoids and vitamins C and E in their antiradical action (77). Phenolic compounds and betalains play an important health-promoting role in human diet, and here we demonstrate that their content in crops can be enhanced by optimizing the light spectrum.

## 5 Conclusion

In this study, we investigated the effects of different red-to-blue (R:B) light ratios on biometrics as well as on antioxidative components in red amaranth and red lettuce. Light ratios influenced the growth of red amaranth and red lettuce, and the accumulation of plant secondary metabolites. Higher proportions of red light resulted in increased stem height in red amaranth and leaf length in red lettuce, with R:B of 9:1 having the strongest effect. The increase of the blue light fraction caused the upregulation of antioxidative components and antioxidant capacity, which reached the highest levels under R:B of 1:1 in most instances. Our findings demonstrate that it is possible to use LED lights in a vertical farm setting to modulate, possibly enhance, the phenotypic properties and/or nutritional quality of crops, using different ratios of red and blue light. Overall, light recipes can be individually tailored according to the type of crop as well as the desired outcomes.

## Data availability statement

The original contributions presented in the study are included in the article/Supplementary material, further inquiries can be directed to the corresponding author.

## Author contributions

AB: Conceptualization, Data curation, Formal analysis, Investigation, Methodology, Project administration, Writing – original draft, Writing – review & editing. MP: Conceptualization, Data curation, Formal analysis, Methodology, Supervision, Writing – original draft, Writing – review & editing. VP: Conceptualization, Methodology, Writing – original draft, Writing – review & editing. SP: Data curation, Formal analysis, Writing – original draft, Writing – review & editing. CA: Data curation, Formal analysis, Writing – original draft, Writing – review & editing. TK: Methodology, Resources, Writing – original draft, Writing – review & editing. GM: Data curation, Supervision, Writing – original draft, Writing – review & editing. AJ: Funding acquisition, Project administration, Supervision, Writing – original draft, Writing – review & editing. DS: Conceptualization, Funding acquisition, Supervision, Writing – original draft, Writing – review & editing.
