# Supplementary material for: Light manipulation as a route to enhancement of antioxidant properties in red amaranth and red lettuce
Source: Front Nutr. 2024 Jun 5;11:1386988. doi: 10.3389/fnut.2024.1386988 (PMC11186462; doi:10.3389/fnut.2024.1386988)
Supplement: Supplementary file 1 [file Data_Sheet_1.docx]

Supplementary Material

Light manipulation as a route to enhancement of antioxidant properties in red amaranth and red lettuce

**Annika Bucky^1*^, Martina Pičmanová^2^, Victoria Porley^3^, Simon Pont^2^, Ceri Austin^2^, Tanveer Khan^3^, Gordon McDougall^2^, Alexandra Johnstone^1^, Derek Stewart^2^**

^1^The Rowett Institute, University of Aberdeen, Aberdeen, United Kingdom

^2^Advanced Plant Growth Centre, The James Hutton Institute, Invergowrie, Dundee, United Kingdom

^3^Intelligent Growth Solutions, Invergowrie, Dundee, United Kingdom

***Correspondence:**Annika Bucky
[a.bucky.22@abdn.ac.uk](mailto:a.bucky.22@abdn.ac.uk)

**Supplementary Table 1.** Biometric measurements in red amaranth and red lettuce grown under different light recipes. Values represent mean ± SD (n = 5). Different letters indicate significant differences among treatments (ANOVA, Tukey’s test, *p* < 0.05).

|  | **Red amaranth** | | **Red lettuce** | |
| --- | --- | --- | --- | --- |
| **Treatment** | **Stem height (cm)** | **Dry weight (%)** | **Leaf length (cm)** | **Dry weight (%)** |
| **RB1** | 1.31 ± 0.09 d | 8.00 ± 0.09 b | 6.49 ± 0.62 c | 6.28 ± 0.25 |
| **RB2.5** | 1.75 ± 0.10 c | 8.78 ± 0.09 a | 7.70 ± 0.48 b | 6.40 ± 0.31 |
| **RB5** | 2.18 ± 0.11 b | 9.17 ± 0.40 a | 8.42 ± 0.68 ab | 6.22 ± 0.43 |
| **RB9** | 2.49 ± 0.17 a | 8.12 ± 0.17 b | 8.96 ± 0.73 a | 6.26 ± 0.32 |

Supplementary Table 2. The quantities of antioxidant components and antioxidant capacity in dry weight of red amaranth and red lettuce under the four light treatments. DW, Dry weight, TPC, Total phenol content, GAE, gallic acid equivalent, C3GE, cyanidin-3-glucoside equivalent. Values represent mean ± SD (n = 5). Different letters indicate significant difference (ANOVA, Tukey’s test, *p* < 0.05).

|  | **Red amaranth** | | | | **Red lettuce** | | | |
| --- | --- | --- | --- | --- | --- | --- | --- | --- |
| **Treatment** | **TPC** | **Betalains** | **Vitamin C** | **FRAP** | **TPC** | **Anthocyanins** | **Vitamin C** | **FRAP** |
|  | (g GAE 100 g^-1^ DW) | (mg 100 g^-1^ DW) | (mg 100 g^-1^ DW) | (mmol Fe^2+^ 100 g^-1^ DW) | (g GAE 100 g^-1^ DW) | (mg C3GE 100 g^-1^ DW) | (mg 100 g^-1^ DW) | (mmol Fe^2+^ 100 g^-1^ DW) |
| **RB1** | 2.20 ± 0.08 a | 936.90 ± 63.40 a | 513.50 ± 25.86 b | 13.54 ± 0.42 a | 6.54 ± 0.45 a | 230.78 ± 34.91 a | 350.53 ± 14.26 b | 52.46 ± 9.22 a |
| **RB2.5** | 2.08 ± 0.08 ab | 854.40 ± 25.39 a | 577.47 ± 19.70 a | 12.18 ± 0.83 ab | 5.85 ± 0.52 ab | 203.14 ± 28.59 ab | 423.11 ± 13.51 a | 39.88 ± 8.09 ab |
| **RB5** | 2.03 ± 0.02 b | 726.20 ± 107.78 b | 545.28 ± 20.04 ab | 11.70 ± 0.53 b | 5.80 ± 0.54 ab | 181.85 ± 21.61 b | 283.36 ± 17.91 c | 43.93 ± 7.21 ab |
| **RB9** | 1.98 ± 0.06 b | 667.40 ± 60.75 b | 331.42 ± 25.66 c | 9.36 ± 1.35 c | 5.60 ± 0.30 b | 157.55 ± 19.65 b | 326.16 ± 9.07 b | 33.55 ± 4.05 b |

Supplementary Table 3. The quantities of antioxidant components and antioxidant capacity in fresh weight of red amaranth and red lettuce under the four light treatments. FW, Fresh weight, TPC, Total phenol content, GAE, gallic acid equivalent, C3GE, cyanidin-3-glucoside equivalent. Values represent mean ± SD (n = 5). Different letters indicate significant difference (ANOVA, Tukey’s test, *p* < 0.05).

|  | **Red amaranth** | | | | **Red lettuce** | | | |
| --- | --- | --- | --- | --- | --- | --- | --- | --- |
| **Treatment** | **TPC** | **Betalains** | **Vitamin C** | **FRAP** | **TPC** | **Anthocyanins** | **Vitamin C** | **FRAP** |
|  | (g GAE 100 g^-1^ FW) | (mg 100 g^-1^ FW) | (mg 100 g^-1^ FW) | (mmol Fe^2+^ 100 g^-1^ FW) | (g GAE 100 g^-1^ FW) | (mg C3GE 100 g^-1^ FW) | (mg 100 g^-1^ FW) | (mmol Fe^2+^ 100 g^-1^ FW) |
| **RB1** | 0.18 ± 0.01 a | 74.95 ± 4.97 a | 41.09 ± 2.20 b | 1.09 ± 0.03 a | 0.41 ± 0.04 a | 14.55 ± 2.61 a | 22.05 ± 1.69 b | 3.30 ± 0.62 a |
| **RB2.5** | 0.18 ± 0.01 a | 75.07 ± 2.66 a | 50.73 ± 1.77 a | 1.07 ± 0.06 a | 0.38 ± 0.05 a | 13.11 ± 2.44 ab | 27.18 ± 2.11 a | 2.58 ± 0.63 ab |
| **RB5** | 0.19 ± 0.01 a | 66.57 ± 10.35 a | 50.04 ± 3.86 a | 1.07 ± 0.07 a | 0.36 ± 0.06 a | 11.42 ± 2.07 ab | 17.72 ± 2.03 c | 2.75 ± 0.56 ab |
| **RB9** | 0.16 ± 0.01 b | 54.19 ± 5.38 b | 26.92 ± 2.48 c | 0.76 ± 0.13 b | 0.35 ± 0.03 a | 9.89 ± 1.57 b | 20.41 ± 1.46 bc | 2.11 ± 0.35 b |


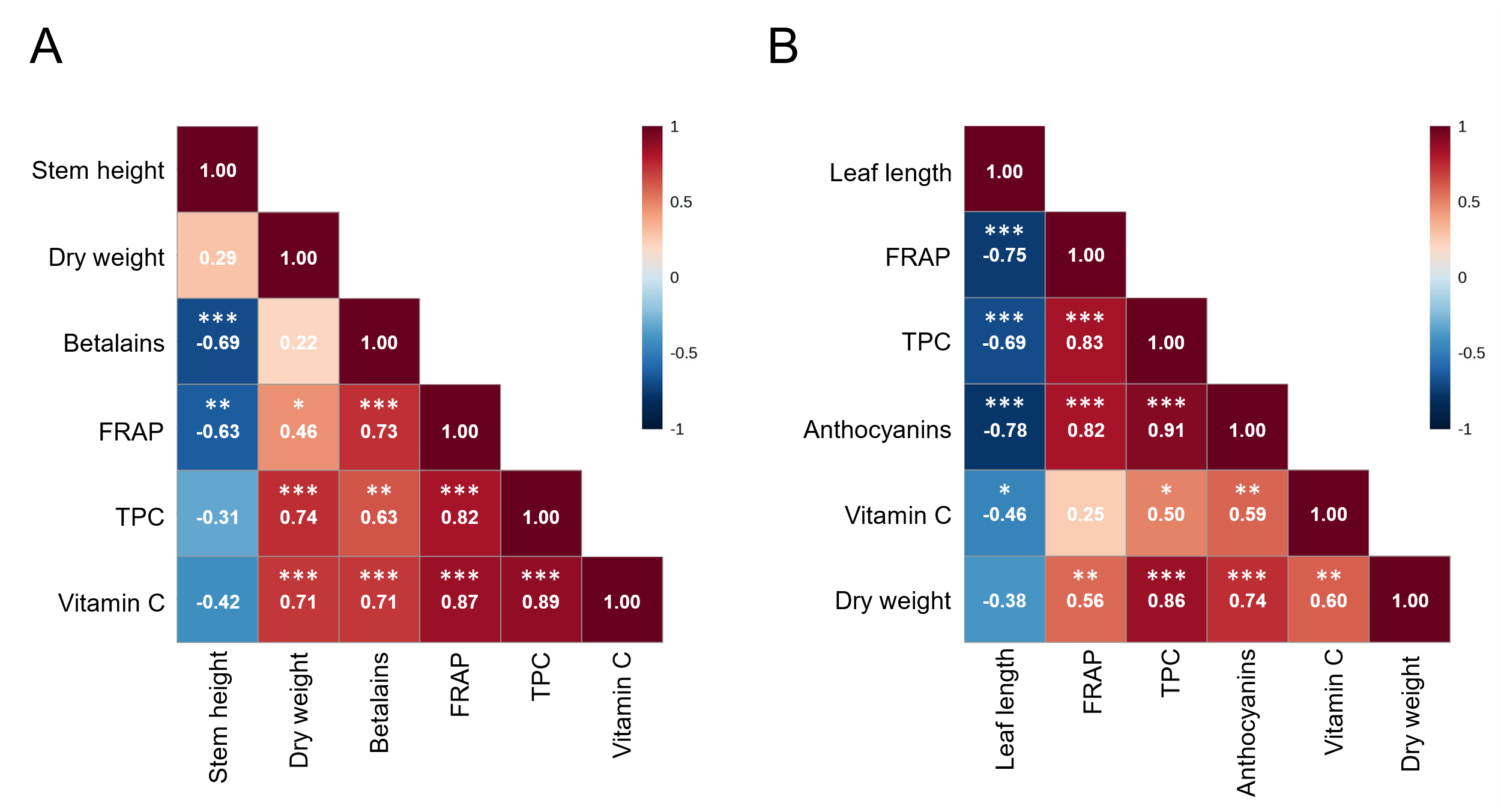


**Supplementary Figure 1.** Correlation heatmaps showing Pearson correlation coefficients between stem height/leaf length, dry weight percentage, total phenol content (TPC), betalains/anthocyanins, vitamin C and FRAP in fresh weight of red amaranth (A) and red lettuce (B), as generated by MetaboAnalyst 5.0. Data were log_10_-transformed and auto-scaled; **p* < 0.05, ***p* < 0.01, ****p* < 0.001.
